# Secular Trends in Preterm Birth Rates: Uncovering the Primary Challenge for Perinatal Medicine in Greece

**DOI:** 10.7759/cureus.67295

**Published:** 2024-08-20

**Authors:** Nikolaos Vlachadis, Dionysios N Vrachnis, Nikolaos Loukas, Nikolaos Antonakopoulos, Alexandros Fotiou, Theodoros Karampitsakos, Panagiotis Anastasopoulos, Georgios Maroudias, Zoi Iliodromiti, Nikolaos Vrachnis

**Affiliations:** 1 Department of Obstetrics and Gynecology, General Hospital of Messinia, Kalamata, GRC; 2 Third Department of Obstetrics and Gynecology, Medical School, National and Kapodistrian University of Athens, Attiko Hospital, Athens, GRC; 3 Department of Obstetrics and Gynecology, Tzaneio Hospital, Piraeus, GRC; 4 Department of Obstetrics and Gynecology, School of Health Sciences, University of Patras, Patras, GRC; 5 Medical School, National and Kapodistrian University of Athens, Athens, GRC; 6 Department of Obstetrics and Gynecology, Iaso Maternity Hospital, Athens, GRC; 7 Department of Obstetrics and Gynecology, Santorini Hospital, Thira, GRC; 8 Neonatal Department, Medical School, National and Kapodistrian University of Athens, Aretaieio Hospital, Athens, GRC

**Keywords:** economic crisis, duration of gestation, pregnancy, births, perinatal medicine, preterm birth rate, premature birth, trend analysis, greece, preterm birth

## Abstract

Introduction: Preterm labour is a serious pregnancy complication that is the primary cause of infant mortality, with detrimental impacts on the offspring and the mother in the short as well as the long term. This study aims to comprehensively present the time trends of national preterm birth rates (PBRs) in Greece.

Methods: Official national data regarding live births in Greece were acquired from the Hellenic Statistical Authority, and the annual total PBR and rates for gestational age groups were computed per 100 total live births spanning from 1980 to 2022. Time trends were analyzed through joinpoint regression analysis, and annual percent changes (APC) and average annual percent change (AAPC) were calculated with a 95% confidence interval (95% CI).

Results: Following a steady decline from 4.66% in 1980 to a historic low of 2.77% in 1991 with an APC of −5.1 (−6.2 to −4.2), the PBR exhibited a dramatic increase during 1991-2011 with an APC of 7.3 (6.9 to 7.8). Subsequently, between 2011 and 2022, the rise in PBR was attenuated, showing a slight statistically non-significant upward trend (APC = 0.5, 95% CI: −0.6 to 1.5). This led to a historical high of 12.07% in 2018, 4.4 times higher than that in 1991, and eventually, the PBR reached 11.90% in 2022. From 1991 to 2022, there were sharper increases in the rates of moderate (32-33 weeks) and late (34-36 weeks) preterm births, with AAPCs of 4.9 (3.5-6.4) and 5.8 (5.3-6.3), respectively. In contrast, the rates of extremely (<28 weeks) and very (28-31 weeks) preterm births saw slower growth, with AAPCs of 2.2 (1.7-2.7) and 0.7 (0.5-1.0), respectively.

Conclusion: The PBR in Greece more than quadrupled during 1991-2022, mainly due to increases in moderate and late preterm births. Although its rise has markedly decelerated since 2011, amidst the country's economic recession, the PBR is alarmingly higher than those in all other European and developed nations. More than one in nine neonates is born prematurely in the Greek population, posing challenges in implementing evidence-based prevention strategies and perinatal care.

## Introduction

The World Health Organization defines preterm birth as any live birth occurring at less than 37 completed gestational weeks, or 259 days from the first day of the mother's last menstrual period. The threshold of 37 weeks for preterm birth is considered to some extent arbitrary and serves to emphasize the distribution of risks within the 40-week gestational range in comparison with full-term pregnancies [1,2]. Preterm birth is a major challenge in obstetrics and perinatal medicine, accounting for about 75% of perinatal deaths and more than half of long-term neonatal health issues. Each year, more than one million newborns die from issues related to preterm birth. It is the leading cause of infant mortality, responsible for a third of the three million annual neonatal deaths worldwide, and the second most frequent cause of mortality in children under the age of five [3-5].

Survival among preterm neonates is associated with an elevated susceptibility to various short-term and delayed health complications. Common issues resulting from premature birth include a heightened occurrence of bronchopulmonary dysplasia and respiratory distress, necrotizing enterocolitis, brain haemorrhage, encephalopathy, and other neurological complications, as well as ophthalmological and auditory impairments [2,6].

Preterm birth ranks as the fourth leading cause globally of human capital loss across all age groups, trailing only ischaemic heart disease, pneumonia, and severe diarrhoea. Moreover, individuals born prematurely face an elevated risk of chronic illnesses during adulthood, which can manifest as metabolic disorders, cardiac complications, significant disabilities, and learning impairments. Thus, preterm labour stands out as a principal factor in disability-adjusted life years and the number of years lost because of early death.

These outcomes contribute to substantial physical, mental, and financial burdens. In 2005, it was estimated that the United States incurred costs exceeding US$ 26 billion in medical expenses, educational expenditures, as well as lost productivity tied to preterm births, a figure that continues to rise. Furthermore, the woman who delivers prematurely may experience serious long-term metabolic complications, including an enhanced risk of cardiovascular mortality, hypertension, diabetes, hyperlipidaemia, and metabolic disorders [1,4,7-9].

Premature birth is classified as extremely (<28 weeks), very (28 to 31 weeks), moderate (32 to 33 weeks), and late (34 to 36 weeks) preterm according to the duration of gestation. Furthermore, preterm birth can be divided into three categories, reflecting different epidemiologies and a distinct etiological spectrum. Around half of the preterm births are considered idiopathic, while 30% are linked to preterm prelabour rupture of membranes (PPROM), and approximately 20% are medically indicated (iatrogenic) [8,10,11].

Approximately 13.4 million live births are preterm, representing 9.9% of all live births worldwide, or 1 in 10 babies. Preterm birth rates (PBRs) vary in different geographic areas, with the most elevated rates observed in Southern Asia (13.2% in 2020) and lower rates in North America and Europe (less than 8%). Despite medical advances, little progress has been made on trends in global preterm birth rates in the 21st century, while there are notable divergences in rates and trends among nations of different income levels and among developed countries [1,12]. Preterm labour is a serious complication of pregnancy that carries major implications for public health. The objective of this study was to present the longitudinal trends in premature birth rates in Greece since 1980 at the national level.

## Materials and methods

National official data regarding live births in Greece, categorized by gestational age (in completed weeks) and based on registered birth certificates, were obtained from the Hellenic Statistical Authority [13]. These data span a 43-year period from 1980 to 2022, the earliest and latest years with accessible information. During this time, there were a total of 4,534,314 live births in Greece. For a small portion of these births, specifically 2,216 (0.05%), gestational age data were not available, and these were proportionally distributed across gestational weeks.

Live births were defined as those occurring after 20 weeks of gestation [14,15], and the PBR was calculated as the number of births at less than 37 gestational weeks per 100 total live births. Additionally, preterm birth rates were further categorized into four sub-groups: extremely preterm birth rate (EPBR), very preterm birth rate (VPBR), moderately preterm birth rate (MPBR), and late preterm birth rate (LPBR) for births occurring before 28, 28-31, 32-33, and 34-36 weeks, respectively. All rates were expressed per 100 live births. The estimation of excess premature births from 1992 to 2022 was carried out by summing the differences between the observed annual preterm births and the number that would have occurred if the 1991 PBR had remained constant.

The Joinpoint Regression Program, version 5.2.0 (National Cancer Institute, United States of America), was utilized to analyze trends in preterm birth rates. The annual percent change (APC) and the average annual percent change (AAPC) were computed with a 95% confidence interval (95% CI), and p < 0.05 was considered the level of statistical significance. An APC was determined for each segment between two joinpoints (years with statistically significant trend change), with a maximum of seven segments allowed, while the AAPC served as a comprehensive measure of the overall trend across a specified period, calculated as a weighted average of the APCs depending on the duration of each segment. Approval from an ethics board was deemed unnecessary, as in this study, publicly available national data were analyzed to maintain individual anonymity.

## Results

**Figure 1:**
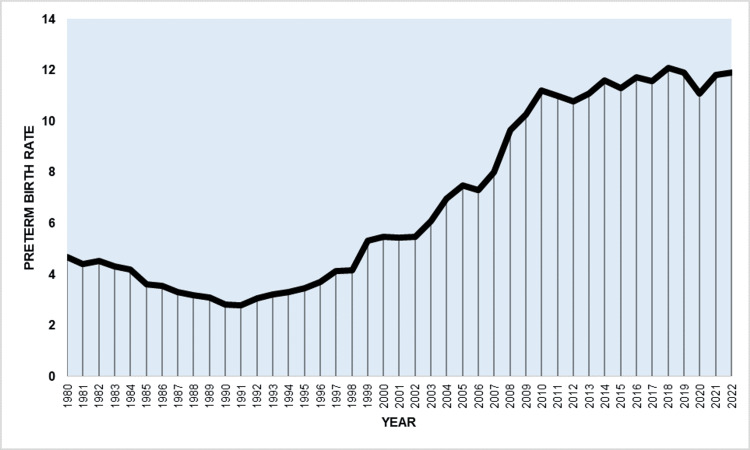
Preterm birth rate (per 100 live births) in Greece, 1980-2022.

**Figure 2:**
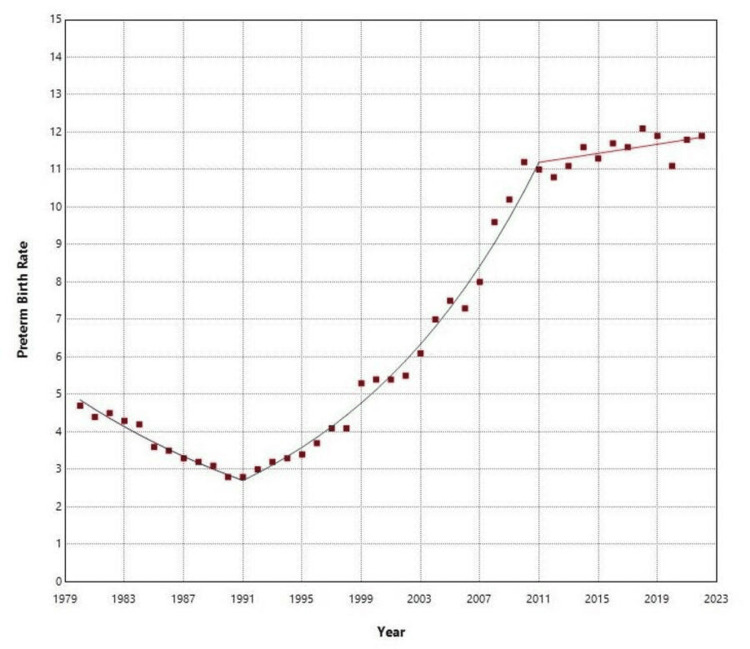
Time trends in preterm birth rates (per 100 live births) in Greece, 1980-2022.

During the period spanning from 1980 to 2022, there were a total of 4,534,314 live births, with 300,668 of them being preterm, resulting in an overall PBR of 6.63%. The PBR decreased by 41%, from 4.66% in 1980 to a low of 2.77% in 1991. From 1980 to 1991, the decline in PBR had an APC of −5.1 (95% CI: −6.2 to −4.2). Subsequently, from 1991 to 2011, there was an upward trend in the PBR with an APC of 7.3 (95% CI: 6.9 to 7.8). Between 2011 and 2022, the PBR remained elevated with a slight statistically non-significant rising trend (APC = 0.5, 95% CI: −0.6 to 1.5), reaching an all-time high of 12.07% in 2018, which was 4.4 times higher compared to 1991. The rate decreased by 8% to 11.05% in 2020 (down 7% during 2019-2020) before rising again to 11.90% in 2022 (Table 1 and Figures 1-2).

**Table 1:** Time trends in preterm birth rates in Greece, 1980-2022.

Segment	Annual percent change	95% confidence interval	P-value
1980–1991	−5.1	−6.2 to −4.2	<0.001
1991–2011	7.3	6.9–7.8	<0.001
2011–2022	0.5	−0.6 to 1.5	0.271

**Figure 3:**
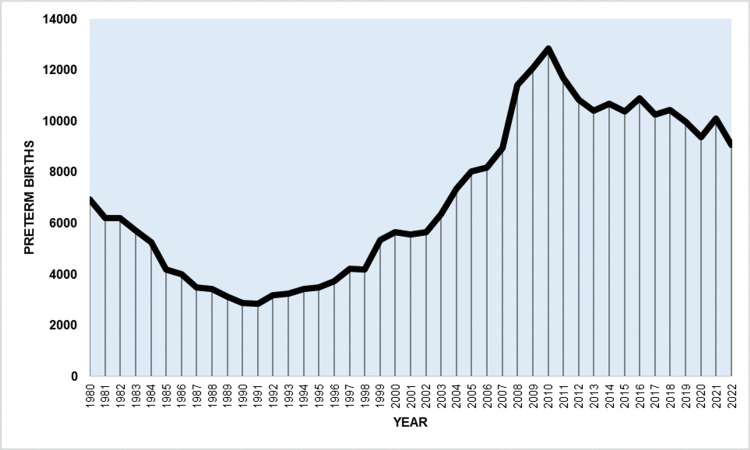
Preterm births in Greece, 1980-2022.

The number of preterm births decreased by 59%, from 6,910 to 2,839 between 1980 and 1991, and then surged 4.5 times to a peak of 12,831 in 2010. Subsequently, from 2010 to 2022, preterm births decreased by 29% to 9,059 (Figure 3).

**Figure 4:**
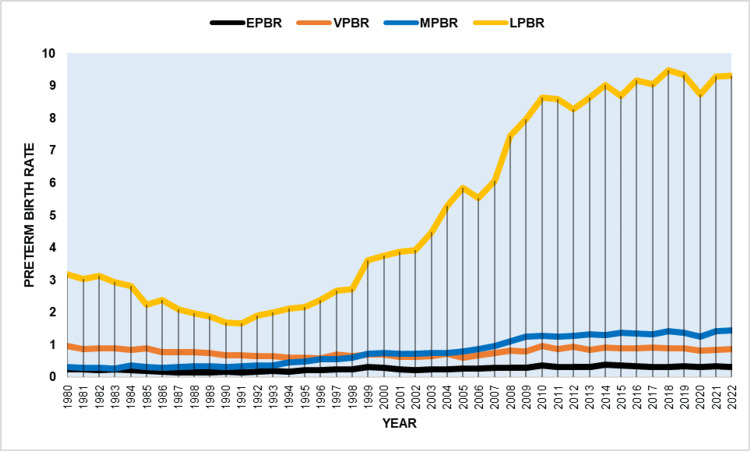
Preterm birth rates (per 100 live births) by gestational age sub-groups in Greece, 1980-2022. EPBR: extremely preterm birth rate (<28 weeks); VPBR: very preterm birth rate (28-31 weeks); MPBR: moderately preterm birth rate (32-33 weeks); LPBR: late preterm birth rate (34-36 weeks).

**Figure 5:**
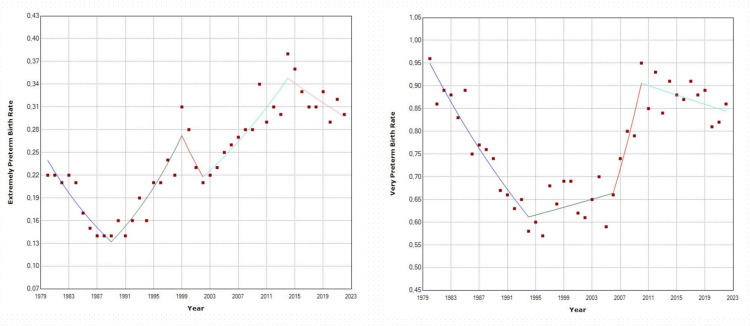
Time trends in extremely preterm birth rates (<28 weeks) and very preterm birth rates (28-31 weeks) (per 100 live births) in Greece, 1980-2022.

The increase in PBR after 1991 resulted in an estimated 160,805 additional preterm births during the period of 1992-2022. Among total preterm births from 1980 to 2022, 73.6% were late preterm, 11.2% were moderately preterm, 11.6% were very preterm, and 3.6% were extremely preterm births. The EPBR demonstrated a notable reduction from 1980 to 1989 (APC = −6.4, 95% CI: −8.8 to −4.3), followed by an increase until 2014 with a slight deviation from 1999 to 2002: 1989-1999: APC = 7.5 (95% CI: 5.6 to 12.2); 1999-2002: APC = −7.2 (95% CI: −10.9 to 1.2); 2002-2014: APC = 4.0 (95% CI: 2.7 to 11.6). Subsequently, from 2014 to 2022, there was a non-significant downward trend (APC = −1.9, 95% CI: −6.3 to 0.4). The EPBR ranged from a low of 0.14% in 1991 to a high of 0.38% in 2014, reaching 0.30% in 2022 (Table 2, Figures 4-5).

**Table 2:** Time trends in extremely preterm birth rates (<28 weeks) in Greece, 1980-2022.

Segment	Annual percent change	95% confidence interval	P-value
1980–1989	−6.4	−8.8 to −4.3	0.006
1989–1999	7.5	5.6 to 12.2	0.022
1999–2002	−7.2	−10.9 to 1.2	0.115
2002–2014	4.0	2.7 to 11.6	0.010
2014–2022	−1.9	−6.3 to 0.4	0.105

The VPBR experienced a decrease from 1980 to 1994 with an APC of −3.1 (95% CI: −4.5 to −2.4) but then showed no significant changes, except for a brief upward trend between 2006 and 2010 (APC = 8.1, 95% CI: 3.3 to 12.5). The variation in VPBR ranged from a maximum of 0.96% in 1980 to a minimum of 0.57% in 1996, and finally, 0.86% in 2022 (Table 3 and Figures 4-5).

**Table 3:** Time trends in very preterm birth rates (28-31 weeks) in Greece, 1980-2022.

Segment	Annual percent change	95% confidence interval	P-value
1980–1994	−3.1	−4.5 to −2.4	0.006
1994–2006	0.7	−1.2 to 2.0	0.334
2006–2010	8.1	3.3 to 12.5	0.015
2010–2022	−0.6	−1.7 to 0.2	0.144

**Figure 6:**
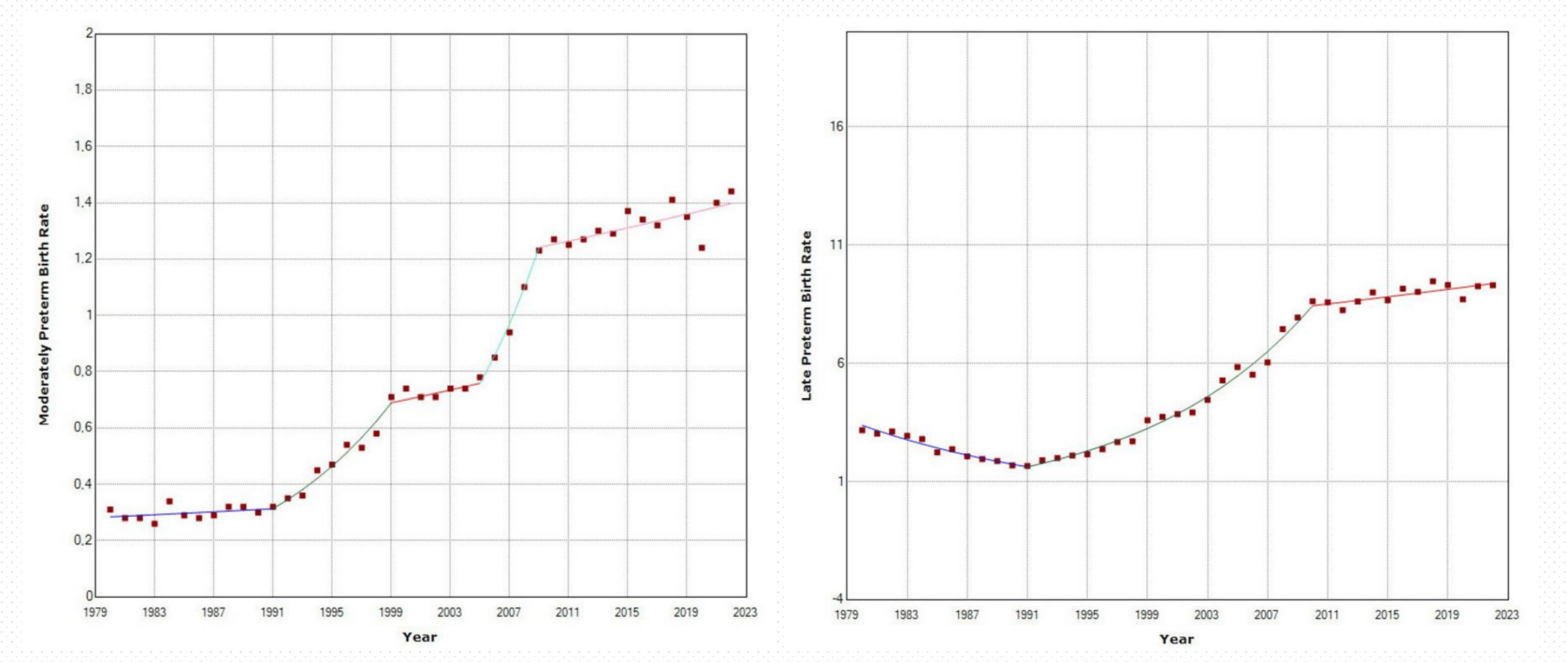
Time trends in moderately preterm birth rates (32-33 weeks) and late preterm birth rates (34-36 weeks) (per 100 live births) in Greece, 1980-2022.

The MPBR decreased to a low of 0.26% in 1983 before showing a non-significant increase from 1980 to 1991, followed by a steep rise until 2010 with an APC of 10.4 (95% CI: 8.7 to 14.3). There was stability in MPBR from 1999 to 2005, after which it rapidly increased until 2009 (APC = 13.1, 95% CI: 8.6 to 18.8) and then continued to rise at an average annual rate of 0.9% until 2022, when it peaked at 1.44% (Table 4 and Figures 4-6).

**Table 4:** Time trends in moderately preterm birth rates (32-33 weeks) in Greece, 1980-2022.

Segment	Annual percent change	95% confidence interval	P-value
1980–1991	0.9	−0.3 to 1.9	0.115
1991–1999	10.4	8.7 to 14.3	<0.001
1999–2005	1.6	−4.1 to 3.9	0.432
2005–2009	13.1	8.6 to 18.8	0.001
2009–2022	0.9	0.1 to 1.6	0.037

The LPBR exhibited a sharp decline from 1980 to a minimum of 1.66% in 1991 with an APC of −6.5 (95% CI: −7.8 to −5.4), followed by a fast increase until 2010 with an APC of 9.1 (95% CI: 8.5 to 9.7). The upward trend in LPBR continued until 2022, reaching a peak of 9.47% in 2018 and 9.30% in 2022 without exceeding the threshold of statistical significance (Table 5 and Figures 4-6).

**Table 5:** Time trends in late preterm birth rates (34-36 weeks) in Greece, 1980-2022.

Segment	Annual percent change	95% confidence interval	P-value
1980–1991	−6.5	−7.8 to −5.4	<0.001
1991–2010	9.1	8.5 to 9.7	<0.001
2010–2022	0.9	−0.2 to 1.9	0.101

Table 6 presents the trends in PBR for different gestational age sub-categories from 1991 to 2022.

**Table 6:** Trends in preterm birth rate sub-categories in Greece, 1991-2022.

Preterm birth rate sub-category	1991–2022 Average annual percent change	95% confidence interval	P-value
Extremely preterm birth rate (<28 weeks)	2.2	1.7–2.7	<0.001
Very preterm birth rate (28–31 weeks)	0.7	0.5–1.0	<0.001
Moderately preterm birth rate (32–33 weeks)	4.9	4.6–5.2	<0.001
Late preterm birth rate (34–36 weeks)	5.8	5.5–6.1	<0.001

**Figure 7:**
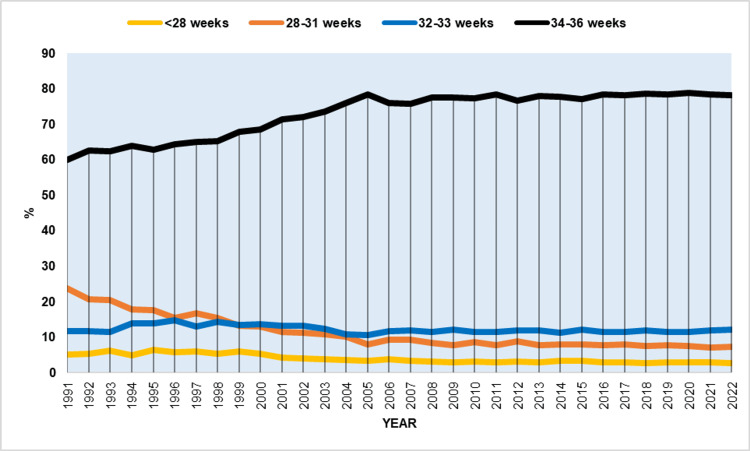
Relative contribution of different gestational age sub-groups in preterm births in Greece, 1991-2022.

Between 1991 and 2022, EPBR and VPBR showed slow but statistically significant increases, with AAPCs of 2.2 (95% CI: 1.7 to 2.7) and 0.7 (95% CI: 0.5 to 1.0), respectively. In contrast, MPBR and LPBR exhibited steep upward trends, with AAPCs of 4.9 (95% CI: 3.5 to 6.4) and 5.8 (95% CI: 5.3 to 6.3), respectively. During this period, MPBR increased by 4.5 times, from 0.32% to 1.44%, and LPBR by 5.6 times, from 1.66% to 9.30%. Consequently, the percentage of very preterm births in relation to total births at less than 37 weeks gestation decreased, from 23.7% to 7.2%, and in extremely preterm births, from 4.9% to 2.5%. In contrast, the percentage of moderately preterm births increased slightly from 11.5% to 12.1%, while the share of late preterm births rose substantially from 59.9% to 78.1% (Figure 7).

The increase in LPBR accounted for 84% of the total increase in PBR from 1991 to 2022. Table 7 presents a detailed analysis of the increasing trends in rates of prematurity at 32-36 weeks by gestational week.

**Table 7:** Time trends in preterm birth rates by gestational week (32-36 weeks) in Greece, 1991-2022.

Gestational age (weeks)	1991–2022 Average annual percent change	95% confidence interval	P-value
32	3.6	3.1–4.2	<0.001
33	6.1	5.5–6.7	<0.001
34	3.5	3.0–3.9	<0.001
35	6.6	6.3–6.9	<0.001
36	6.2	5.8–6.5	<0.001

**Figure 8:**
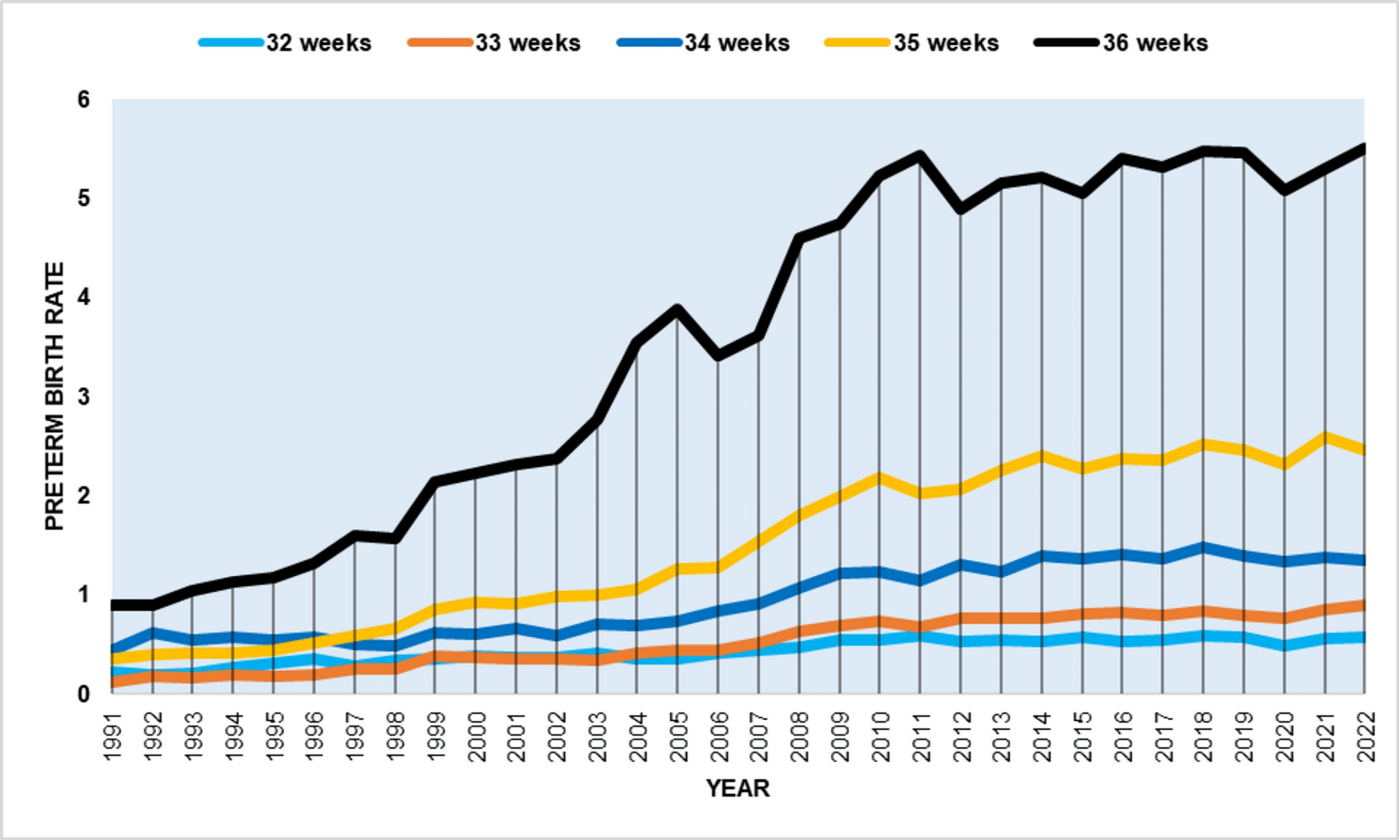
Preterm birth rates by gestational week (per 100 live births) (32-36 weeks) in Greece, 1991-2022.

The data show that preterm births increased at an average annual rate of more than 6% at 35 and 36 weeks, as well as at 33 weeks. However, the rate of increase was slower at 32 and 34 gestational weeks. Between 1991 and 2022, the PBR at 36 weeks increased from 0.89% to 5.50%, representing a 6.2-fold increase. The share of preterm births at 36 weeks among all births <37 weeks also rose from 32.1% to 46.2%. Additionally, the PBR at 35 weeks increased from 0.34% to 2.46%, a 7.3-fold increase, while the rate at 33 weeks climbed from 0.11% to an all-time high of 0.89%, representing a 7.9-fold increase (Figure 8).

## Discussion

The current research utilized official data at the national level to showcase the secular patterns in premature birth rates in Greece spanning more than four decades. The results indicate a steep rise in prematurity rates in Greece during the 1990s and 2000s. Post-2010, premature birth rates remained elevated, albeit with a flatter slope at the limits of statistical significance. The best-fitting model from the joinpoint analysis identified three sub-periods for PBR trends. During the initial sub-period spanning from 1980 to 1991, the PBR experienced a notable decline of 41%, with an average annual decrease of 5.1%, ultimately reaching a historic low of 2.77%. The most significant decreases were observed in the EPBR and LPBR, while the VPBR saw a lesser decline and the MPBR remained statistically unchanged. The improvement in PBR throughout the 1980s was undeniably linked to the advancements in living standards and socioeconomic conditions, as well as in antenatal care and access to health services facilitated by the establishment of the national health system in Greece [16].

The declining trend observed from 1980 to 1991 was completely reversed in the subsequent two decades (1991-2011), during which the PBR increased more than fourfold, with an average annual growth rate of 7.3%. Subsequently, the upward trajectory halted in the final period from 2011 to 2022, where the PBR exhibited a slight, statistically non-significant increase and reached a record peak of more than 12 premature births per 100 live births in 2018. From 1991 to 2022, steeper rises were seen in LPBR and MPBR, by 5.6 and 4.5 times, respectively, with lesser increases in VPBR and EPBR. A more detailed analysis by gestational week revealed the largest increases at 36, 35, and 33 weeks, with an average annual rate exceeding 6%.

Global data indicate a rising trend in the global PBR from 1990 to 2010, with minimal change thereafter. The PBR was consistently higher in low-income countries and lower in developed nations, with significant differences in rates and trends observed across various countries. In 2005, approximately 12.9 million births were premature worldwide, accounting for 9.6% of all births. The highest prevalence of preterm births was in Africa and North America, at 11.9% and 10.6%, respectively, while it was lower at 6.2% in Europe. In 2010, the global PBR increased to 11.1% and preterm births to 14.9 million. In developed countries, there was an increase in the average PBR from 7.2% in 1990 to 8.6% in 2010 [4,8].

In 2010, the estimated global PBR was 9.8%. This figure rose to 10.6% by 2014, with rates of prematurity varying from 8.7% in Europe to 13.4% in North Africa [2]. According to a recent analysis, around 13.4 million neonates were born preterm in 2020, representing 9.9% of total births, without significant alteration in global PBR between 2010 and 2020. In 2020, sub-Saharan Africa combined accounted for more than 65% of global preterm births. Between 2010 and 2020, roughly 15% of total premature births occurred before 32 gestational weeks worldwide [12].

Preterm births resulting from spontaneous preterm labour and PPROM are viewed as a syndrome with a multifactorial aetiology. Although approximately 50% of the causes remain unidentified, various pathologic mechanisms have been proposed, including infection, inflammation, stress, placental insufficiency, hormonal imbalances, vascular abnormalities, cervical dysfunction, and immunological maternal-foetal interaction [7,10].

Internationally, primary risk factors for preterm labour include multiparity and short interpregnancy intervals, low socioeconomic status, inadequate nutrition, chronic maternal systemic diseases and infections, and limited access to healthcare, resulting in insufficient antenatal care. Notably, in developed nations, the surge in prematurity rates is primarily linked to a rise in indicated preterm births, which usually stem from major pregnancy complications, including pre-eclampsia and foetal growth restriction. The escalation of preterm births is also influenced by the high occurrence of preterm multiple gestations due to assisted reproductive technology (ART) treatments. Noteworthy, singleton pregnancies following in vitro fertilization also exhibit heightened susceptibility to preterm birth [2,4,5,7,10-12,17-19].

The rising trend of preterm births in Greece may be associated with three key factors: advancing maternal age, higher rates of multiple pregnancies linked to the growing use of ART services, and an increasing incidence of induced preterm labour by healthcare providers. Over recent decades, the Greek population has seen a notable surge in births among older women. Between 1990 and 2010, there were sizeable increases of 180% and 250% in births for the 35-39 and 40-44 age groups, respectively [20]. Another study reported increases in the preterm birth risk from 1999 to 2008 by 54% for the 35-39 age group and by 90% for women aged 40-44 years [21].

The results of the present study show that the significant increase in preterm births in Greece was mainly associated with increases in late and moderately preterm births. The MPBR reached a historical peak of 1.44% in 2022, with a notable increase observed for births at 33 weeks gestation, potentially linked to the concurrent increase in multiple gestations. Furthermore, in 2022, the proportion of late preterm births increased to 78.1%, and the PBR at 36 weeks rose by a factor of 6.2, accounting for 46.2% of all preterm births. This escalation may suggest a rise in medically indicated preterm births.

In Greece, the PBR and the multiple birth rate have increased since 1990 [22]. A significant positive correlation between the increase in the multiple birth rate in 19 European countries and the rise in PBR from 1996 to 2008 was also noted in 19 European countries (Spearman’s rho = 0.66). In 2008, the proportion of the overall PBR attributable to multiple births ranged from around 17% to more than 27% in various countries [23]. The data from Greece revealed a strong positive correlation between the rise in PBR and that in multiple birth rates during the same timeframe (rho = 0.956), significantly exceeding the correlations observed in the other European countries, whereas in 2008, the population risk of preterm birth attributable to multiple births was calculated to be 25.7% [24].

The sharp incline in prematurity rates in Greece was attenuated after 2011, a period coinciding with the intensification of the unprecedented economic downturn affecting the country. This trend shift could potentially be linked to a decrease in the utilization of ART by Greek couples due to financial constraints or alterations in clinical practices related to the transfer of multiple embryos [22,25]. Furthermore, there was a significant decline in PBR by 7% from 2019 to 2020, marking the most substantial annual decrease since 1980, potentially associated with a further decrease in the utilization of ART treatments in the first year of the coronavirus disease epidemic [26].

In 2013, a descriptive study utilizing national data revealed a sizeable surge in premature births in Greece between 1991 and 2008, primarily attributed to a rise in late preterm births. This study marked the first instance in the literature where trends in preterm births were identified as a growing concern for the country [16]. Subsequent advanced statistical analysis demonstrated that from 1991 to 2010, the PBR in Greece followed an almost perfect exponential growth (R2 = 0.983), with the rate doubling on average every 9.5 years [27]. The growth rates of PBR in Greece were found to be the highest among European countries from 1996 to 2008 [20], as well as among countries with the highest human development index from 1999 to 2010 [28]. This enormous increase has led to Greece having the highest prematurity rates among all developed countries globally. A comparison of Greece's PBR with data from the Euro-Peristat information system that evaluates perinatal rates in the European Union (EU) (not including data from Greece) revealed that in 2019, Greece had the highest PBR among all EU countries [29]. Additionally, an analysis conducted by Ohuma et al. indicated that in 2020, Greece held the highest PBR among 54 countries across North America, Europe, Central Asia, Australia, and New Zealand [12].

The dramatic rise of Greece to the top position among the world's most advanced health countries poses a major challenge for perinatal medicine in the country. It is undoubtedly encouraging that this rise primarily affected births beyond 31 weeks of gestation. Besides, high rates of prematurity between 32 and 36 weeks are prevalent in nations with advanced obstetric care and may be linked to reduced rates of stillbirth and neonatal mortality [30]. Nevertheless, late preterm infants continue to be linked to significant health and economic challenges compared with full-term births [31].

Research indicates that variations in the magnitude of PBR in high-income nations are influenced by reproductive health policies and medical procedures, such as fertility treatments and elective deliveries [32]. Essential steps to decrease PBR in Greece should involve the diligent adoption of evidence-based protocols and policies focused on decreasing elective preterm births, which have demonstrated notable decreases in the rates of provider-initiated premature deliveries. Notably, around half of electively inducted preterm deliveries may have occurred without clear medical justification [4,33]. Additionally, modifications in medical practices in ART clinics are required to minimize the occurrence of multiple pregnancies. Finally, efforts should be made to promote equal access to prenatal care services and implement early screening and prevention measures for pregnancies at risk [3,34].

The strengths of the present study involve the utilization of official nationwide data obtained from birth certificates over a period of more than four decades, allowing for a thorough examination of temporal patterns and trends as well as the specific analysis of PBR by gestational age. Conversely, the constraints of the current analysis stem from challenges in accurately determining gestational age, which is crucial in defining preterm birth. Gestational age can be reliably estimated in pregnancies resulting from in vitro fertilization based on the transferred embryo’s age and in spontaneous pregnancies from the first day of the last menstrual period (LMP) in conjunction with early ultrasound assessment. Nonetheless, accuracy may significantly fluctuate in irregular menstrual cycles, unknown LMPs, or cases of first ultrasound evaluation in later stages of pregnancy [16].

## Conclusions

The current examination of longitudinal trends revealed the epidemic scale of the rising prevalence of PBR in Greece, which holds the highest PBR among economically developed nations. These findings pose dramatic challenges for perinatal medicine in the country, particularly considering adverse shifts in sociodemographic and obstetric factors linked with premature labour, including advancing maternal age and a high burden of multiple pregnancies. It is crucial to promptly implement preventive interventions that prioritize health equity and a critical approach towards elective preterm labour and ART practices.

## References

[1] World Health Organization (2024). Born too soon: the global action report on preterm birth. https://iris.who.int/handle/10665/44864.

[2] Chawanpaiboon S, Vogel JP, Moller AB (2019). Global, regional, and national estimates of levels of preterm birth in 2014: a systematic review and modelling analysis. Lancet Glob Health.

[3] (2021). Prediction and prevention of spontaneous preterm birth: ACOG practice bulletin, number 234. Obstet Gynecol.

[4] Blencowe H, Cousens S, Oestergaard MZ (2012). National, regional, and worldwide estimates of preterm birth rates in the year 2010 with time trends since 1990 for selected countries: a systematic analysis and implications. Lancet.

[5] Chang HH, Larson J, Blencowe H (2013). Preventing preterm births: analysis of trends and potential reductions with interventions in 39 countries with very high human development index. Lancet.

[6] Iliodromiti Z, Anastasiadis A, Varras M (2013). Monocyte function in the fetus and the preterm neonate: immaturity combined with functional impairment. Mediators Inflamm.

[7] Romero R, Dey SK, Fisher SJ (2014). Preterm labor: one syndrome, many causes. Science.

[8] Beck S, Wojdyla D, Say L (2010). The worldwide incidence of preterm birth: a systematic review of maternal mortality and morbidity. Bull World Health Organ.

[9] Crump C, Sundquist J, Howell EA, McLaughlin MA, Stroustrup A, Sundquist K (2020). Pre-term delivery and risk of ischemic heart disease in women. J Am Coll Cardiol.

[10] Goldenberg RL, Culhane JF, Iams JD, Romero R (2008). Epidemiology and causes of preterm birth. Lancet.

[11] Walani SR (2020). Global burden of preterm birth. Int J Gynaecol Obstet.

[12] Ohuma EO, Moller AB, Bradley E (2023). National, regional, and global estimates of preterm birth in 2020, with trends from 2010: a systematic analysis. Lancet.

[13] (2024). Hellenic Statistical Authority. https://www.gov.gr/en/arxes/ellenike-statistike-arkhe-elstat.

[14] Vlachadis N, Siori M, Petrakos G (2023). Tracing time trends of births in Greece. Cureus.

[15] Vlachadis N, Vrachnis D, Antonakopoulos N (2023). Temporal trends in stillbirth in Greece: a longitudinal population-based study. Cureus.

[16] Baroutis G, Mousiolis A, Mesogitis S, Costalos C, Antsaklis A (2013). Preterm birth trends in Greece, 1980-2008: a rising concern. Acta Obstet Gynecol Scand.

[17] Malamitsi-Puchner A, Vrachnis N, Samoli E (2006). Investigation of midtrimester amniotic fluid factors as potential predictors of term and preterm deliveries. Mediators Inflamm.

[18] Malamitsi-Puchner A, Vrachnis N, Samoli E, Baka S, Hassiakos D, Creatsas G (2006). Elevated second trimester amniotic fluid interferon gamma-inducible T-cell alpha chemoattractant concentrations as a possible predictor of preterm birth. J Soc Gynecol Investig.

[19] Tsikouras P, Dafopoulos A, Trypsianis G (2012). Pregnancies and their obstetric outcome in two selected age groups of teenage women in Greece. J Matern Fetal Neonatal Med.

[20] Vlachadis N, Vrachnis N, Tsikouras P, Mastorakos G, Iliodromiti Z (2015). Birth rates by maternal age in Greece: background, trends and future perspectives. J Reprod Med.

[21] Mousiolis A, Baroutis G, Sindos M, Costalos C, Antsaklis A (2013). Maternal age as a predictive factor of pre-term birth. An epidemiological study from 1999 to 2008 in Greece. J Obstet Gynaecol.

[22] Vlachadis N, Vrachnis D, Loukas N (2023). Temporal trends in multiple births in Greece: the evolution of an epidemic. Cureus.

[23] Zeitlin J, Szamotulska K, Drewniak N (2013). Preterm birth time trends in Europe: a study of 19 countries. BJOG.

[24] Vlachadis N, Iliodromiti Z, Creatsas G, Vrachnis N (2014). Preterm birth time trends in Europe: the worrying case of Greece. BJOG.

[25] Sfakianoudis K, Simopoulou M, Rapani A (2019). The impact of the economic recession in Greece on assisted reproduction demand: a retrospective longitudinal study. Medicina (Kaunas).

[26] Scaravelli G, Levi Setti PE, Gennarelli G (2022). The actual impact of SARS-CoV-2/COVID-19 pandemic on IVF activity: a survey across Italian ART centers. J Assist Reprod Genet.

[27] Vlachadis N, Kornarou E, Ktenas E (2013). The preterm births epidemic in Greece. Acta Obstet Gynecol Scand.

[28] Vlachadis N, Kornarou E (2013). Preterm births in countries with a very high human development index. Lancet.

[29] (2024). The European perinatal health report, 2015-2019. https://www.europeristat.com/index.php/reports/ephr-2019.html.

[30] Lisonkova S, Sabr Y, Butler B, Joseph KS (2012). International comparisons of preterm birth: higher rates of late preterm birth are associated with lower rates of stillbirth and neonatal death. BJOG.

[31] Loftin RW, Habli M, Snyder CC, Cormier CM, Lewis DF, DeFranco EA (2010). Late preterm birth. Rev Obstet Gynecol.

[32] Delnord M, Blondel B, Zeitlin J (2015). What contributes to disparities in the preterm birth rate in European countries?. Curr Opin Obstet Gynecol.

[33] Bouchet N, Gayet-Ageron A, Lumbreras Areta M, Pfister RE, Martinez de Tejada B (2018). Avoiding late preterm deliveries to reduce neonatal complications: an 11-year cohort study. BMC Pregnancy Childbirth.

[34] Reddy M, McGannon C, Mol BW (2024). Looking back on preterm birth - the successes and failures. Acta Obstet Gynecol Scand.

